# Cryoballoon Pulmonary Vein Isolation in Obese Patients with Atrial Fibrillation Compared to Non-Obese Counterparts: A Meta-Analysis

**DOI:** 10.3390/biomedicines13020298

**Published:** 2025-01-25

**Authors:** Dimitrios A. Vrachatis, Konstantinos A. Papathanasiou, Dimitrios Kazantzis, Ioannis Anagnostopoulos, Maria Kousta, Sotiria G. Giotaki, Gerasimos Deftereos, Vaia Lambadiari, George Giannopoulos, Efthimia K. Basdra, Theodore G. Papaioannou, Gerasimos Siasos, Spyridon Deftereos

**Affiliations:** 1 Eugenideio Hospital, National and Kapodistrian University of Athens, 11528 Athens, Greece; dvrachatis@gmail.com (D.A.V.); iannis.anagnostopoulos@gmail.com (I.A.); mkousta@med.uoa.gr (M.K.); sotiria.giotaki@yahoo.com (S.G.G.); gerasimosd@gmail.com (G.D.); ebasdra@med.uoa.gr (E.K.B.); spdeftereos@gmail.com (S.D.); 2 Department of Biomedical Engineering, National and Kapodistrian University of Athens, Medical School, 11527 Athens, Greece; teogpap@gmail.com; 3 Second Department of Cardiology, National and Kapodistrian University of Athens, Medical School, Attikon University Hospital, 12462 Athens, Greece; 4 NIHR Moorfields Biomedical Research Centre and Clinical Research Facility, Moorfields Eye Hospital NHS Foundation Trust, London EC1V 2PD, UK; dkaza91@yahoo.gr; 5 Second Department of Internal Medicine, National and Kapodistrian University of Athens, Medical School, Attikon University Hospital, 12462 Athens, Greece; vlambad@otenet.gr; 6 Third Department of Cardiology, Aristotle University of Thessaloniki, 54124 Thessaloniki, Greece; ggiann@med.uoa.gr; 7 Department of Biological Chemistry, National and Kapodistrian University of Athens, Medical School, 12462 Athens, Greece; 8 Third Department of Cardiology, National and Kapodistrian University of Athens, Medical School, Sotiria Chest Disease Hospital, 11527 Athens, Greece; gsiasos@med.uoa.gr

**Keywords:** atrial fibrillation, obesity, body mass index, catheter ablation, arrhythmia recurrence, radiation exposure

## Abstract

**Introduction:** Obesity is an important risk factor for atrial fibrillation (AF) development. Data on cryoballoon ablation (CBA) outcomes in obese patients have so far been scarce. We reviewed the existing literature to compare the efficacy and safety of CBA in obese versus non-obese AF patients. **Methods:** A systematic literature search was conducted for studies comparing clinical outcomes (arrhythmia recurrence and/or procedural data and/or safety outcomes) between obese and non-obese patients undergoing CBA for AF. Statistical pooling was performed according to a random-effects model with generic inverse-variance weighting of relative risks (RRs) and standardised mean differences (SMDs) computing risk estimates with 95% confidence intervals (CIs). **Results:** Obese and non-obese patients had comparable arrhythmia recurrence rates (normal versus overweight, RR = 0.95, 95% CI: 0.82–1.11, *p* = 0.55, I^2^% = 91%; normal versus class I obesity, RR = 0.97, 95% CI: 0.82–1.13, *p* = 0.68, I^2^% = 87%; normal versus class II obesity, RR = 0.98, 95% CI: 0.91–1.07, *p* = 0.29, I^2^% = 65%). Procedure time was marginally increased in obese patients compared to non-obese counterparts (normal versus overweight, SMD = 0.05, 95% CI: −0.15–0.26, *p* = 0.62, I^2^% = 74%; normal versus class I obesity, SMD = 0.10, 95% CI: −0.00–0.19, *p* = 0.06, I^2^% = 2%; overweight versus class I obesity, SMD = 0.11, 95% CI: 0.01–0.21, *p* = 0.048, I^2^% = 25%). Regarding radiation exposure, fluoroscopy time was increased in patients with class I obesity compared to normal-weight or overweight patients and dose area product was also increased in obese patients compared to non-obese patients. Lastly, the risk of complications did not differ between obese and non-obese patients. Statistical heterogeneity and the small number of patients included are the main limitations of this study. **Conclusion:** CBA seems to be effective for obese patients suffering from AF, featuring also similar safety outcomes with non-obese individuals. Radiation exposure was increased in obese patients.

## 1. Introduction

Atrial fibrillation (AF) is the most common arrhythmia in clinical practice affecting 44 million people worldwide [1]. Obesity is causally associated with AF development [2,3], and meta-analyses have shown that, for every five-unit increase in body mass index (BMI), the relative risk of AF development increases by ≈25% [4,5]. A recent study utilising data from UK Biobank indicated a strong and direct association between increased BMI and AF development [6].

An increasing number of obese patients are undergoing pulmonary vein isolation (PVI), since ≈30% of the patients with paroxysmal AF (PAF) from the ESC-EHRA AF ablation long-term registry featured a BMI > 30 kg/m^2^. Nevertheless, most previously published randomised controlled trials (RCTs) did not report on the impact of obesity on AF ablation safety and efficacy [7]. European registries have indicated that radiofrequency ablation (RFA) for AF in patients with increased BMI is associated with worse outcomes and reduced arrhythmia-free survival [8,9]. On the contrary, a contemporary series of overweight and obese patients with AF undergoing high-power short-duration PVI indicated that the procedure is safe and effective [10].

Although overweight and obese patients undergoing ablation should be strongly encouraged to lose weight before the procedure [11,12], highly symptomatic patients might have difficulties in complying with exercise programs. The Obesity Reduction Trial for AF Ablation Patients (SORT-AF) was a RCT that evaluated the impact of a dedicated weight control program for patients with AF and obesity (BMI 30–40 kg/m^2^). In this trial, AF burden was not affected by weight control after 12 months of follow-up, and 30% of the participants had poor adherence to the program [13].

Cryoballoon ablation (CBA) is a safe and effective ablation modality [14], and accumulating evidence supports its role in early rhythm control for PAF patients [15,16,17]. The impact of obesity on CBA clinical outcomes needs further research. In this systematic review, we aimed to explore the impact of BMI on the safety, the procedural parameters, and the clinical efficacy of CBA in AF patients.

## 2. Methods

### 2.1. Data Sources and Search Strategy

Studies including patients undergoing CBA for AF in which clinical outcomes such as arrhythmia recurrence, procedural data, and complications were reported and compared between obese and non-obese patients were examined for inclusion in this review. Instructions described in the Preferred Reporting Items for Systematic Reviews and Meta-Analyses 2009 guidelines were followed [18]. All relevant studies were identified by two independent reviewers (K.P. and D.V.) through an electronic search (from inception to 7th of December 2024) of the following databases: MEDLINE database, Scopus, ClinicalTrials.gov, medRxiv, and Cochrane Library. The following search query was used: “atrial fibrillation cryoballoon ablation” or “atrial fibrillation cryoablation” or “atrial fibrillation ablation” and “obesity” or “overweight” or “body mass index” or “BMI” ( Supplementary Table S1). The reference lists of the identified articles were examined in order to identify further eligible studies, articles, and book chapters. In addition, the identified studies were reviewed manually so as to exclude duplicates.

### 2.2. Inclusion and Exclusion Criteria

In order for a study to be eligible, it had to fulfil the following inclusion criteria: (1) evaluated arrhythmia recurrence and/or procedural data and/or complications in patients undergoing CBA, (2) employed clearly stated definitions of obesity and arrhythmia recurrence, and (3) reported outcome data separately for obese and non-obese patients. Studies were excluded if they (1) were case reports or (2) evaluated the above-stated outcomes in other ablation modalities for AF (RFA, pulse field ablation, laser ablation, surgical, epicardial, or hybrid ablation).

### 2.3. Data Extraction

Two independent reviewers (K.P. and D.V.) extracted and reviewed the data. Any discrepancy between them was addressed through a discussion or by a third reviewer (D.K.). Data such as first author, publication year, study design, study period, number of patients, baseline population characteristics, descriptive statistics and matching criteria for obese and non-obese patients, AF recurrence, complications, and procedural data such as, procedure time, fluoroscopy time, left atrial dwell time, dose area product, freeze duration, and number of freezes were collected.

### 2.4. Quality Assessment

We used the Newcastle–Ottawa scale (NOS) to assess the quality of the studies that were included in our analyses [19]. According to the pre-specified criteria, all studies were evaluated for their adequacy and representativeness in terms of selection, comparability, and exposure/outcome, and a nine-point scale was computed accordingly. Two authors (K.P. and D.K.) were in charge of the implementation of the NOS assessment tool and independently examined the studies. Studies scoring more than seven points were defined as high-quality, those between four and six points as moderate-quality, and ultimately those with less than four points as poor-quality.

### 2.5. Outcomes of Interest

The pre-specified primary endpoint was arrhythmia recurrence between obese and non-obese patients post-CBA. The secondary endpoints were complications and procedural differences.

### 2.6. Statistical Analysis

We pooled study-specific data with the inverse-variance random-effects method, and continuous variables were expressed as standardised mean difference (SMD) with 95% confidence intervals (CIs). If the mean and/or the standard deviation (SD) was not available, they were derived from sample size, median, and interquartile range [20]. Furthermore, categorical variables were expressed as a percentage, and the relative risk (RR) with 95% CI was computed accordingly. We assessed for statistical heterogeneity among studies via the Cochran Q chi-square test with *p* ≤ 0.1 considered to be of statistical significance. Tau-square was used to evaluate the variance of the true effect sizes across the included studies, and the I^2^ test was used as a measure of heterogeneity and inconsistency. Publication bias was examined through the inspection of the funnel plots. Review Manager version 5.3 (Copenhagen: The Nordic Cochrane Centre, The Cochrane Collaboration, 2014) was used for the analyses, and the guidelines presented in the MOOSE statement were followed [21].

## 3. Results

### 3.1. Search Results

**Figure 1:**
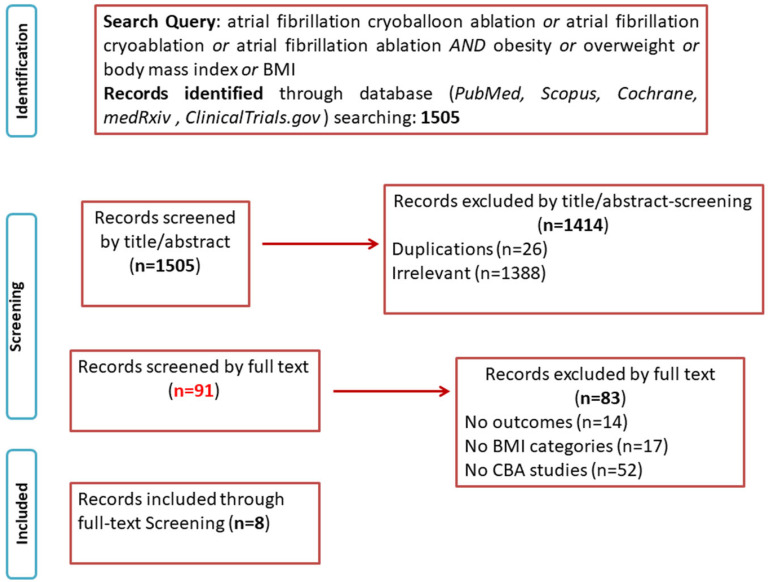
PRISMA flow chart.

The study search and selection process are depicted in Figure 1. The electronic database search identified 1505 studies. After screening, a total of eight studies [22,23,24,25,26,27,28,29] met the inclusion criteria. Since the majority of the included studies reported CBA outcomes for normal weight, overweight, and obese AF patients, we opted to exclude two studies that dichotomised their population into patients with a BMI < 30 kg/m^2^ and a BMI > 30 kg/m^2^ [30,31]. Individual study characteristics are presented in Table 1. Definitions regarding obesity and AF recurrence, as well as exclusion criteria and periprocedural management details, are provided in Supplementary Table S2. Cryoablation protocol and procedural data are described in Supplementary Table S3.

### 3.2. Arrhythmia-Free Survival

A total of five studies compared AF recurrence between normal weight and overweight or class I obesity patients and found that arrhythmia-free survival is comparable across these BMI categories (normal weight vs. overweight, RR = 0.95, 95% CI: 0.82–1.11, *p* = 0.55, I^2^% = 91%, and normal weight vs. class I obesity, RR = 0.97, 95% CI: 0.82–1.13, *p* = 0.68, I^2^% = 87%).

**Figure 2:**
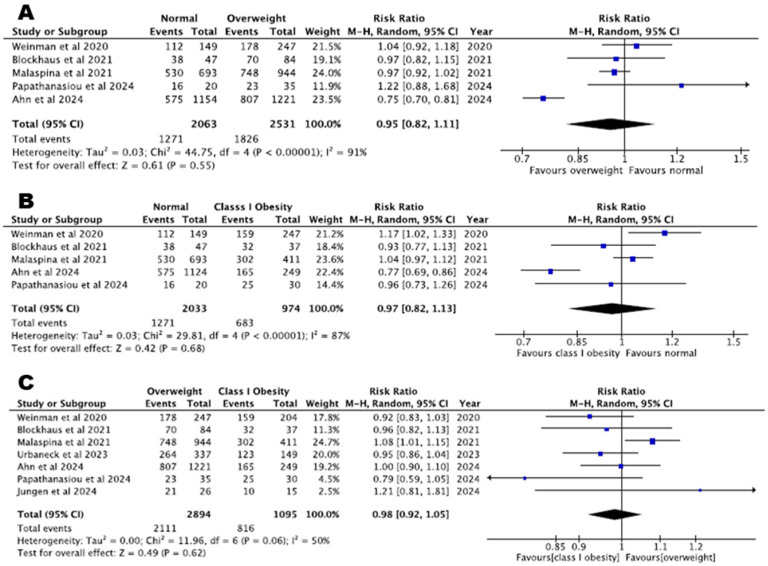
Arrhythmia-free survival: (**A**) normal BMI versus overweight patients, (**B**) normal BMI versus class I obesity patients, (**C**) overweight versus class I obesity patients [22,23,24,26,27,28,29].

Seven studies including 3989 patients undergoing CBA found that arrhythmia-free survival does not differ between overweight and class I obesity patients (RR = 0.98, 95% CI: 0.92–1.05, *p* = 0.62, I^2^% = 50%). Arrhythmia-free survival across patients with different BMI classes is depicted in Figure 2.

### 3.3. Procedural Data

#### 3.3.1. Total Procedure Time

**Figure 3:**
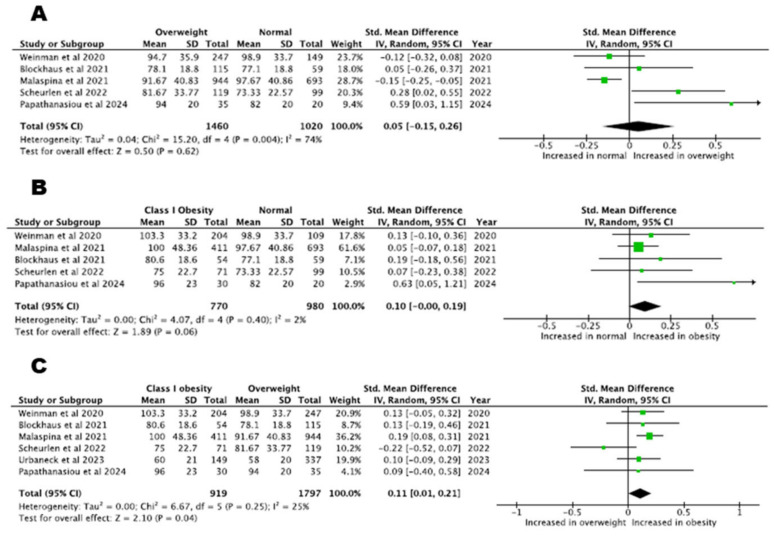
Procedure time: (**A**) normal BMI versus overweight patients, (**B**) normal BMI versus class I obesity patients, (**C**) overweight versus class I obesity patients [22,23,24,25,29].

In total, six studies compared procedure time between normal weight and overweight or class I obesity and found an increasing trend in higher-BMI categories (normal weight vs. overweight patients, five studies with 2480 patients, SMD= 0.05, 95% CI: −0.15–0.26, *p* = 0.62, I^2^% = 74%, normal weight vs. class I obesity patients, five studies with 1750 patients, SMD = 0.10, 95% CI: −0.00–0.19, *p* = 0.06, I^2^% = 2%). The procedure time in patients with class I obesity was increased compared to overweight patients (SMD = 0.11, 95% CI: 0.01–0.21, *p* = 0.048, I^2^% = 25%, six studies). Procedure time across patients with different BMI classes is depicted in Figure 3.

We were not able to pool data regarding patients with class II obesity and normal weight, since only one study provided relevant data. Comparison of the procedure time between class II obesity and overweight patients showed that the time was increased in the former group (two studies, SMD = 0.22, 95% CI: 0.04–0.41, *p* = 0.02, I^2^% = 0%). Lastly, the procedure time was similar in patients with class II obesity compared with patients with class I obesity (two studies, SMD = 0.22, 95% CI: −0.04–0.49, *p* = 0.10, I^2^% = 26%).

#### 3.3.2. Fluoroscopy Time

**Figure 4:**
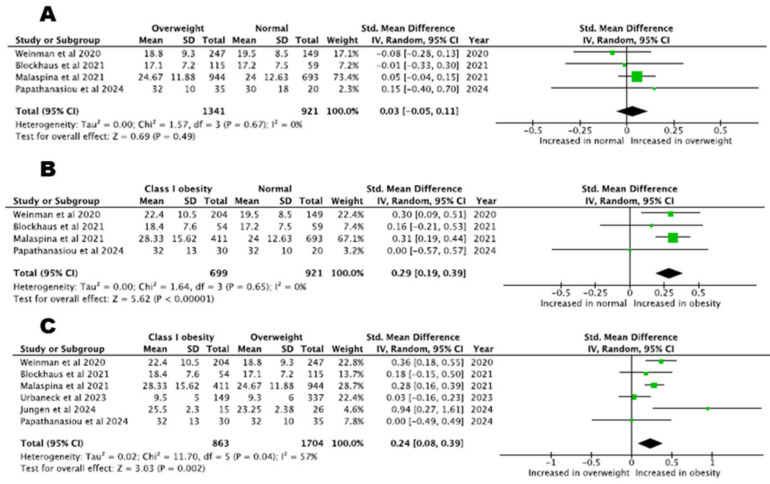
Fluoroscopy time: (**A**) normal BMI versus overweight patients, (**B**) normal BMI versus class I obesity patients, (**C**) overweight versus class I obesity patients [22,23,24,26,28,29].

In total, four studies involving 2262 patients compared fluoroscopy time between normal weight and overweight patients and found no significant differences (SMD = 0.03, 95% CI: −0.05–0.11, *p* = 0.49, I^2^% = 0%). Fluoroscopy time was increased in patients with class I obesity compared to normal weight or overweight patients (class I obesity vs. normal weight patients, four studies with 1620 patients, SMD = 0.29, 95% CI: 0.19–0.39, *p* < 0.00001, I^2^% = 0%, class I obesity vs. overweight patients, six studies with 2567 patients, SMD = 0.24, 95% CI: 0.08–0.39, *p* = 0.002, I^2^% = 57%). Fluoroscopy time across patients with different BMI classes is depicted in Figure 4.

#### 3.3.3. Dose Area Product

**Figure 5:**
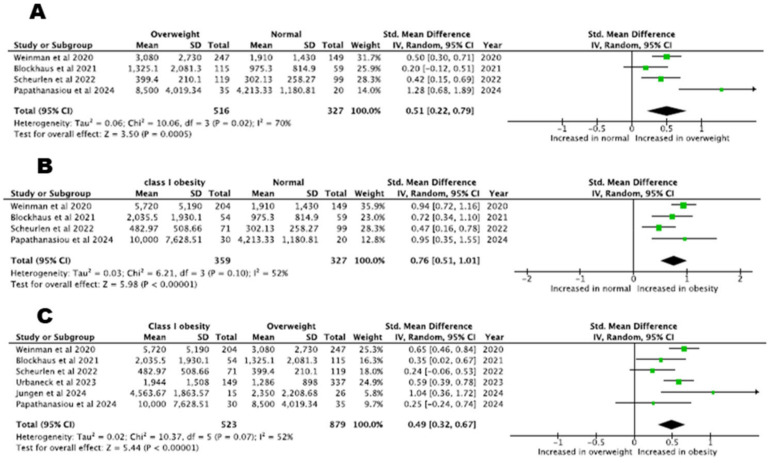
Dose area product: (**A**) normal BMI versus overweight patients, (**B**) normal BMI versus class I obesity patients, (**C**) overweight versus class I obesity patients [22,23,25,26,28,29].

Dose area product (DAP) is a laboratory measurement that assesses exposure to ionising radiation during CBA. A statistically significant trend of increased DAP was observed across patients with increased BMI. Specifically, four studies compared DAP between normal weight and overweight patients (SMD = 0.51, 95% CI: 0.22–0.79, *p* = 0.0005, I^2^% = 70%), four studies compared DAP between normal weight and class I obesity patients (SMD = 0.76, 95% CI: 0.51–1.01, *p* < 0.00001, I^2^% = 52%), six studies compared DAP between overweight and class I obesity patients (SMD = 0.49, 95% CI: 0.32–0.67, *p* < 0.00001, I^2^% = 52%), two studies compared DAP between overweight and class II obesity patients (SMD = 1.33, 95% CI: 1.12–1.53, *p* < 0.00001, I^2^% = 0%), and, lastly, two studies compared DAP between class I and class II obesity (SMD = 0.71, 95% CI: 0.42–0.99, *p* < 0.00001, I^2^% = 32%). DAP across patients with different BMI classes is depicted in Figure 5.

### 3.4. Safety Data

**Figure 6:**
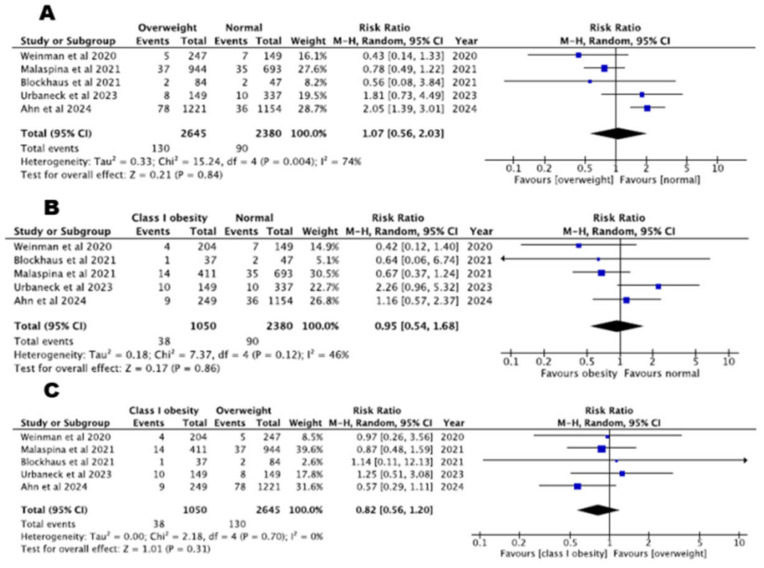
Complications: (**A**) normal BMI versus overweight patients, (**B**) normal BMI versus class I obesity patients, (**C**) overweight versus class I obesity patients [22,23,24,26,27].

A total of five studies found similar complication rates between normal weight and overweight or class I obesity patients (normal weight versus overweight: RR = 1.07, 95% CI: 0.56–2.03, *p* = 0.84, I^2^% = 74%, normal weight versus class I obesity: RR = 0.95, 95% CI: 0.54–1.68, *p* = 0.86, I^2^% = 48%). Furthermore, complications did not differ between patients with class I obesity and overweight patients (five studies with 3695 patients, RR = 0.82, 95% CI: 0.56–1.20, *p* = 0.31, I^2^% = 0%). Complications across patients with different BMI classes are depicted in Figure 6.

### 3.5. Risk of Bias Assessment

The quality assessment scores of the NOS are shown in Table 1 and Supplementary Table S4. Three studies were of moderate quality, while the remaining five were of high quality.

### 3.6. Sensitivity Analysis

As far as arrhythmia-free survival is concerned, the study by Urbaneck et al. was a source of heterogeneity when we compared outcomes between class I obesity and overweight patients. They reported lower AF recurrence rate in overweight as compared to normal weight and obese patients. The study by Ahn et al. was also a source of statistical heterogeneity when we compared outcomes between overweight and normal weight patients. BMI cut-off points differ between Asia–Pacific and Caucasian patients. Nevertheless, the results remained unchanged after excluding these studies from arrhythmia-free survival analyses.

Regarding fluoroscopy time and DAP, the results remained unchanged after the conduction of several leave-one-out sensitivity analyses. The study by Scheurlen et al. was a source of statistical heterogeneity when we compared DAP between class I obesity and normal weight patients. In addition, the study by Urbaneck et al. was a source of statistical heterogeneity when we compared complications between class I obesity and normal weight patients, but this did not affect the results. They found a numerically higher rate of vascular complications at the access site among obese patients.

Finally, regarding procedure time, the study by Scheurlen et al. was a significant source of statistical heterogeneity when we compared outcomes between class I obesity and overweight patients. They found increased procedure time in overweight patients as compared to obese patients. After excluding this study, procedure time was found increased in obese patients undergoing CBA (SMD = 0.16, 95% CI: 0.07–0.24, *p* = 0.0003, I^2^ = 0%). When we compared procedure time between class I obesity and normal weight patients, the studies by Papathanasiou et al. and Blockhaus et al. were sources of heterogeneity, but these studies did not affect the results.

## 4. Discussion

In our meta-analysis, we evaluated eight published studies involving 6570 AF patients and found that increased BMI does not affect arrhythmia-free survival post-CBA. Fluoroscopy time and radiation exposure (dose area product) were significantly increased in obese patients as compared to normal-weight patients. Both total procedure time and complication rates were comparable across different BMI groups. To the best of our knowledge, this is the first meta-analysis to evaluate the impact of BMI on CBA outcomes.

From a clinical perspective, weight control should be strongly encouraged in obese patients, especially in view of its positive impact on arrhythmia-free survival post-ablation [32]. Retrospective data from the US have shown that weight reduction before CBA is associated with reduced late AF recurrence, irrespective of AF subtype and baseline BMI [33]. Since obese patients are frequently unwilling to comply with exercise programs [13] and early rhythm control is associated with increased arrhythmia-free survival, CBA could be an effective and safe approach for overweight and class I obesity individuals. Furthermore, obese patients with AF have impaired quality of life and worse AF-related symptoms as compared to non-obese counterparts. Quality of life is significantly improved post-ablation in obese patients with AF [29,34,35,36].

We found that BMI does not affect arrhythmia-free survival post-CBA. Although previous studies have found that obese patients with AF undergoing RFA have an excess risk of AF recurrence [4], this might be due to unadjusted comorbidities such as hypertension, diabetes mellitus, and sleep apnoea [34]. Also, procedural complications remain a major concern in obese patients undergoing AF ablation. Previous studies focusing mainly on RFA and class I and II obesity patients have found comparable complication rates with non-obese patients [36,37]. Our findings for CBA-treated obese patients are in agreement with these data. Of note, AF patients with morbid obesity (BMI >40 kg/m^2^) might be at increased risk for complications [38,39,40]. Increased BMI is associated with increased femoral vein depth and, consequently, a higher number of unsuccessful cannulation attempts [41]. In a large series of AF patients undergoing CBA, the implementation of ultrasound-guided venipuncture was associated with a significant reduction in major vascular complications [42]. Recently, Urbaneck et al. found that CBA is feasible in class III (morbid) obesity patients, yet the reported that one-year recurrence rate was higher compared to normal weight patients [43].

A previous meta-analysis focusing on RFA-treated AF patients found that total procedure time is increased in obese patients as compared to non-obese counterparts [44]. Nevertheless, no clear explanation was given. In this meta-analysis, we found no difference regarding procedure time between obese and non-obese patients undergoing CBA. We have previously suggested that total procedure time might be prolonged in patients with increased BMI due to the longer time needed to gain venous access [29].

Radiation exposure during AF catheter ablation is far from negligible [45], and the estimated lifetime risk of fatal malignancy development is 0.1% [46]. Previous studies have shown that CBA is associated with increased fluoroscopy time as compared to RFA [45,47] and that increasing BMI is associated with increasing DAP in CBA-treated patients [47]. Although fluoroscopy time is strongly correlated with DAP, there is substantial inter- operator and intraoperator variability in the observed DAP, which implies that the latter is also affected by technical settings [45].

As far as technical settings are concerned, previous studies have suggested that both fluoroscopy time and DAP can be significantly reduced by decreasing the frame rate to 3/sec, avoiding non-selective pulmonary vein angiography and cine angiography [48,49]. Following the radiation safety principles, the Hispanic Systematic Workflow and Electro- gram guidance to reduce X-ray Exposure Time (SWEET-Cryo) strategy for CBA was proven to be both cost-effective and beneficial in terms of radioprotection (reduced DAP exposure) [50].

Cohorts have shown that the use of intracardiac echocardiography (ICE) is associated with reduced DAP [51] and fluoroscopy time [52,53] in AF patients undergoing CBA. A recently published RCT confirmed the above-mentioned findings [54]. However, this trial did not include obese patients, and larger studies addressing the cost-effectiveness of ICE are needed. A novel three-dimensional mapping system (KODEX EPD, EPD-solutions; Philips, Netherlands) has been developed in order to reduce both radiation exposure and iodinated contrast media volume in CBA-treated patients with promising early findings [55].

Previous studies focusing on RFA have shown that obese patients are more likely to suffer from AF recurrence post-ablation [8,56]. To date, no RCT has compared RFA as opposed to CBA in view of radiation exposure in obese patients with AF. In view of radiation exposure, whether cryothermy is a reasonable energy source for AF ablation in obese patients is a matter of debate. Obese patients are commonly affected from comorbidities such as diabetes mellitus, coronary artery disease, various malignancies, and venous thromboembolism, all of which might lead to radiation exposure for diagnostic and therapeutic purposes. Furthermore, AF patients, irrespective of BMI, would possibly need >1 ablation procedure in their lifetime, which increases radiation exposure even further [57].

## 5. Limitations

There were some limitations in our study. First, this is not a patient-level meta- analysis; thus, the impact of several variables, such as baseline demographics, on treatment effects cannot be assessed. Second, the results of this meta-analysis were based on a relatively small number of cohorts with a limited number of obese patients. Furthermore, comparability across BMI categories might be inadequate, since adjustment for potential confounders was not considered in the majority of the included studies. Third, a mixed population of paroxysmal and persistent AF patients was included in the original cohorts, and a subgroup analysis taking into account the AF subtype was not possible. Fourth, our findings regarding the efficacy and safety of CBA in AF patients with increased BMI might not be applicable to patients with class III or morbid obesity, since only a few studies included such BMI categories. Lastly, moderate to high statistical heterogeneity was observed, which might imply methodology issues, such as diverse population characteristics and varying CBA procedure protocols (see Supplementary Tables S2 and S3).

## 6. Conclusions

CBA seems to be a reasonable therapeutic modality for obese and overweight patients suffering from AF, featuring acceptable efficacy and safety profile as compared to normal weight individuals. On the contrary, exposure to ionising radiation is significantly increased in obese patients, and further studies are needed to establish effective radioprotection strategies in this population.

## Figures and Tables

**Table 1:** Studies’ main characteristics.

Author	Study Type	N	AF Type	FU	Female	Age (Years)	DM	AAD Protocol	FU Protocol	NOS
Weinman 2020 (Germany)	Prospective Cohort	600	PAF+ PersAF	24 months	43%	66.3 ± 10.8	18%	NA	12-lead ECG 7-day Holter	6
Blockhaus 2021 (Germany)	Retrospective cohort	228	PAF+ PersAF	12 months	29.8%	NA	NA	NA	12-lead ECG 24 h Holter	7
Malaspina 2021 (Italy)	Retrospective cohort	2048	PAF+ PersAF	10.4 months (3.1–24.5)	27.7%	59.8 ±10.6	5.8%	NA	12-lead ECG 24 h Holter	7
Scheurlen2022 (Germany)	Retrospective cohort	320	PAF+ PersAF	12 months	37%	64.9 ±11.4	10%	NA	12-lead ECG 24 h Holter	7
Urbaneck 2023 (Germany)	Retrospective cohort	600	PAF+ PersAF	12 months	41%	66 ± 11	13%	NA	72 h Holter PPM interrogation	6
Ahn 2024 (Korea)	Retrospective cohort	2648	PAF+ PersAF	610 days (379–865)	23.3%	62 (56–68)	21.5%	NA	12-lead ECG 24 h Holter	7
Jungen 2024 (Germany)	Retrospective cohort	41	PAF+ PersAF	185 days (121–456)	27%	65 (58–74)	32%	Interruption	24 h Holter	7
Papathanasiou 2024 (Greece)	Prospective cohort	85	PAF	12 months	40%	60 ± 10	17.6%	3 months’ allowance	12-lead ECG 24 h Holter	6

N: study population, AF: atrial fibrillation, AAD: antiarrhythmic drug, PAF: paroxysmal atrial fibrillation, PersAF: persistent atrial fibrillation, FU: follow-up, DM: diabetes mellitus, NOS: Newcastle–Ottawa scale, NA: not available, ECG: electrocardiogram, PPM: permanent pacemaker.

## Data Availability

No new data were created or analyzed in this study. Data available upon reasonable request.

## Supplementary Materials

The following are available online at https://www.mdpi.com/article/10.3390/biomedicines13020298/s1, Table S1: Search strings, Table S2: Definitions, exclusion criteria, and anticoagulation management, Table S3: Cryoablation procedure, Table S4: Study quality according to Newcastle–Ottawa scale (NOS). Studies were defined as high-quality if they had more than seven points, as medium-quality if they had between four and six points, and as poor-quality if they had fewer than four points.
