# Supplementary material for: Cryoballoon Pulmonary Vein Isolation in Obese Patients with Atrial Fibrillation Compared to Non-Obese Counterparts: A Meta-Analysis
Source: Biomedicines. 2025 Jan 25;13(2):298. doi: 10.3390/biomedicines13020298 (PMC11852532; doi:10.3390/biomedicines13020298)
Supplement: Supplementary file 1 [file biomedicines-13-00298-s001.zip › biomedicines-3419296-supplementary.pdf]

Supplementary Table S1: Search Strings

Database	Keywords/Search String	Filters	Results
**PubMed**	(atrial fibrillation cryoballoon ablation) OR (atrial fibrillation cryoablation) OR (atrial fibrillation ablation) AND (obesity) OR (overweight) OR (body mass index) OR (BMI)	none	610
**Cochrane Library**	body mass index AND atrial fibrillation	MeSH	405
**medRxiv**	atrial fibrillation ablation AND body mass index	cardiovascular medicine	241
**ClinicalTrials.gov**	atrial fibrillation AND body mass index	none	94
**Scopus**	atrial fibrillation ablation AND body mass index	NOT INDEX MEDLINE	155

Supplementary Table S2: Definitions, exclusion criteria and anticoagulation management

Author	Obesity Classification (Kg/m^2)	TEE Before PVI	Periprocedural OAC	Post-procedural management	Exclusion criteria	AF recurrence (definition)
Weinman 2020 (Germany)	18.5-24.9<br>25-29.9<br>≥30	Yes	uninterrupted VKA<br>NOACs d/c'd 24h prior	Pericardial effusion excluded by echocardiography<br>NOACs were re-administered the day of the procedure<br>OAC was continued for at least 2 mo or longer depending on CHA_2DS_2-VASc score	LA >55 mm<br>uncontrolled HF (NYHA IV)<br>severe valvular disease	any episode of AF, atrial tachycardia, or atrial flutter lasting > 30 s
Blockhaus 2021 (Germany)	<25<br>25-29.9<br>≥30	Yes	uninterrupted VKA<br>NOAC d/c'd 24h prior	Pericardial effusion excluded by echocardiography<br>NOACs were re-administered the day of the procedure<br>OAC was continued for at least 3 mo or longer depending on $CHA_2DS_2VAS_C$ score	N/R	any episode of AF, atrial tachycardia, or atrial flutter lasting > 30 s
Malaspina 2021 (Italy)	<25<br>25-29.9<br>≥30	N/R	N/R	N/R	N/R	any episode of AF, atrial tachycardia, or atrial flutter lasting > 30 s
Scheurlen 2022 (Germany)	<25<br>25-29.9<br>30-35<br>>35	Yes	uninterrupted VKA<br>NOAC d/c'd 24h prior	Pericardial effusion excluded by echocardiography<br>NOACs were re-administered the day of the procedure.	prior LA ablation	any episode of AF, atrial tachycardia, or atrial flutter lasting > 30 s
Urbaneck 2023 (Germany)	25-29<br>30-34<br>≥35	N/R	uninterrupted VKA<br>NOAC d/c'd 24h prior	N/R	N/R	any episode of AF, atrial tachycardia, or atrial flutter lasting > 30 s

Ahn 2024 (Korea)	<18.5<br>18.5-23<br>23-25<br>25-30<br>>30	N/R	N/R	N/R	N/R	any episode of AF, atrial tachycardia, or atrial flutter lasting > 30 s
Jungen 2024 (Germany)	25-30<br>>30	Yes	uninterrupted VKA<br>NOAC d/c'd 24h prior	Pericardial effusion excluded by echocardiography<br>NOACs were re-administered the day of the procedure	ablation beyond PVI except for cavotricuspid isthmus ablation	any episode of AF lasting > 30 s
Papathanasiou 2024 (Greece)	18.5-25<br>25-30<br>>30	Yes	uninterrupted VKA<br>NOAC d/c'd 24h prior	N/R	non-paroxysmal AF,<br>redo ablation,<br>LA >50 mm,<br>primary electrical or structural heart disease	any episode of AF lasting > 30 s
N/R: not reported, TEE: transesophageal echocardiogram, PVI: pulmonary vein isolation, OACs: anticoagulants, VKA: vitamin K antagonists, NOACs: non-vitamin K OACs, d/c'd: discontinued, h: hours, mo: months, s: seconds, AF: atrial fibrillation, LA: left atrium						

Supplementary Table S3: Cryoablation procedure

Author	Site & Date	Redo PVI	Ablation catheter	Freeze protocol and PVI endpoints	Esophageal temperature monitoring	Phrenic nerve stimulation monitoring	Sedation protocol
Weinman 2020 (Germany)	Single center<br><br>01/2013 to 11/2018	No	28-mm balloon Arctic Front Advance & Arctic Front Advance ST (Medtronic, US)	freeze cycle of 240s + bonus cycle or TTI guided protocol<br><br>Entry & Exit block	Yes<br><br>(Sensitherm; St. Jude Medical Inc, St Paul, MN, USA or S-Cath; Circa Scientific Inc., USA)	CMAP + palpation of diaphragm	Deep sedation
Blockhaus 2021 (Germany)	Single center<br><br>01/2018 to 12/2019	N/R	28-mm balloon 2nd gen, Arctic Front Advance (Medtronic, Fridley, MN, US)	freeze cycle of 240s<br><br>Endpoint N/R	Yes (Sensitherm; St. Jude Medical, St Paul, MN, USA)	palpation of diaphragm	Deep sedation
Malaspina 2021 (Italy)	Multi-center<br><br>03/2012 to 10/2018	N/R	Arctic Front or Arctic Front Advance (Medtronic, Inc, Minneapolis, MN, US)	Freeze protocol N/R<br><br>Entry block	N/R	N/R	N/R
Scheurlen 2022 (Germany)	Single center	No	Arctic Front Advance Pro™ (Medtronic, Dublin, Ireland)	.<br>freeze cycle determined by either the observed	Yes (S-Cath, Circa	palpation of diaphragm	Deep sedation

	2018 to 09/2019			TTI or the achieved nadir temperature<br>Entry block	Scientific Inc., Englewood, Colorado, USA)		
Urbaneck 2023 (Germany)	Single center<br>10/2010 to 05/ 2019	N/R	28-mm balloon	freeze cycle of 240s no bonus freeze if TTI<75 s<br>Endpoint N/R	Yes (Sensitherm, St. Jude Medical, Inc)	CMAP + palpation of diaphragm	Deep sedation
Ahn 2024 (Korea)	Multi-center<br>Dates N/R	N/R	28-mm balloon 2nd-gen, Arctic Front Advance (Medtronic)<br>.	Freeze protocol and endpoints were upon the operator's discretion	N/R	palpation of diaphragm	General anesthesia upon the operator's discretion
Jungen 2024 (Germany)	Single center 07/2020 to 03/2023	N/R	28 mm POLARx (Boston Scientific, Massachusetts, US)	Freeze cycle of 180s<br>entrance and exit block	N/R	palpation of diaphragm	N/R
Papathanasiou 2024 (Greece)	Single center Dates N/R	No	28 mm Arctic Front Advance (Medtronic, Inc, Minneapolis, MN, US)	N/R	No	palpation of diaphragm	Deep sedation
N/R: not reported, CMAPs: compound motor action potentials, s: seconds, gen: generation, TTI: time to isolation, PVI: pulmonary vein isolation,							

,

Supplementary Table S4: Study quality according to Newcastle-Ottawa scale (NOS). Studies were defined as high quality if they had more than seven points, as medium quality if they had between four and six points, and as poor quality if they had fewer than four points.

**Author/ Year**	**Journal**	**Selection**	**Comparability**	**Outcome**	**Total Score (quality)**
Weinman 2020	Int J Cardiol	4	0	2	6 (moderate)
Blockhaus 2021	Rev Cardiovasc Med	4	0	3	7 (high)
Malaspina 2021	Pacing Clin Electrophysiol	4	0	3	7 (high)
Scheurlen 2022	J Interv Card Electrophysiol	4	0	3	7 (high)
Urbaneck 2023	J Cardiovasc Electrophysiol	4	0	2	6 (moderate)
Ahn 2024	Europace	4	0	3	7 (high)
Jungen 2024	Int J Cardiol Heart Vasc	4	0	3	7 (moderate)
Papathanasiou 2024	Clin Pract	4	0	2	6 (moderate)
